# Tranexamic Acid Associated With Reduced Postoperative Transfusion Rates in Hip Fracture Surgery: A Retrospective Study

**DOI:** 10.7759/cureus.100678

**Published:** 2026-01-03

**Authors:** Ellen Geary, Gerard A Sheridan, Kealan Blake, Patrick Carroll, Jonathan O'Toole, Jeffrey Kirwan, Emer Scanlon, Parnell Keeling, Conor Hurson

**Affiliations:** 1 Orthopaedic Surgery, St. Vincent's University Hospital, Dublin, IRL; 2 Trauma and Orthopaedics, St. Vincent's University Hospital, Dublin, IRL; 3 Orthopaedics and Trauma, St. Vincent's University Hospital, Dublin, IRL

**Keywords:** hip fracture, safety, thromboembolic, tranexamic acid, transfusion

## Abstract

Aims

In hip fracture surgery, blood transfusions are common. Allogenic blood transfusions can be associated with adverse effects, and so their use should be reduced where possible. The current study aims to further the knowledge base around the use of tranexamic acid (TXA) in hip fracture surgery patients. This study will focus on outcomes following the use of perioperative TXA in hip fracture patients, with the primary aim of assessing transfusion rates and thromboembolic events.

Methods

This was a single-centre, retrospective cohort study performed in a high-volume academic trauma unit managing 416 hip fractures per year. Patients undergoing surgery for a hip fracture between August 1, 2019, and August 1, 2020 were included. Two groups, based on whether or not the patient received TXA, were identified. Primary outcomes of interest were transfusion rates, postoperative haemoglobin levels, and the rate of venous thromboembolism (VTE).

Results

A total of 351 patients were included, 178 in the control group and 173 in the TXA group. On univariate analysis, there were four variables associated with postoperative transfusion: age (RR 1.03, 95% CI 1.01-1.04, p < 0.0001), American Society of Anaesthesiologists (ASA) (RR 1.75, 95% CI 1.35-2.27, p < 0.001), TXA (RR 0.64, 95% CI 0.45-0.94, p = 0.022), and preoperative haemoglobin level (RR 0.93, 95% CI 0.90-0.94, p < 0.0001). On multivariate analysis, both the preoperative haemoglobin (p < 0.0001) and administration of TXA (p = 0.047) were significantly associated with a reduced need for postoperative transfusions. Day 1 haemoglobin levels in the TXA group were significantly higher compared to the no-TXA group (107 g/L, s = 19.9, 95% CI 103.1-109.8 vs 101 g/L, s = 19.2, 95% CI 97.9-104.1, p = 0.0183). With regards to VTE, there was no statistically significant increase in the rate of VTE with the use of TXA for deep venous thrombosis (DVT) (p = 0.242) or pulmonary embolism (PE) (p = 0.242).

Conclusion

Administering intraoperative TXA is associated with reduced postoperative transfusion rates and improved postoperative haemoglobin levels in hip fracture patients, while not increasing VTE events. Further research in this field should focus on determining an ideal dose, mode of delivery, and timing of TXA administration to optimise its efficacy.

## Introduction

Optimising the care of patients with hip fractures to enable early mobilisation and independence, whilst reducing morbidity and mortality, is an ongoing challenge for the international orthopaedic community. There is an increasing incidence of hip fractures globally, with the elderly, osteoporotic population being most affected [1]. Projections have estimated that the number of hip fractures globally will rise from 1.6 million in 2000 to 6.3 million in the year 2050 [1]. Assuming a stable incidence rate, annual hip fracture numbers are expected to increase by 100% in 2026 [2].

In hip fracture surgery, postoperative blood transfusions are commonly reported to be as high as 28.3% [3]. The majority of hip fractures occur in the elderly population with multiple co-morbidities, making them more predisposed to intraoperative blood loss and postoperative anaemia [4]. Anaemia is associated with an increase in morbidity and mortality [5]. Hence, it is important that patients are optimised preoperatively to minimise blood loss [4]. Allogenic blood transfusions only partially solve the problem of intraoperative blood loss, as they are associated with adverse effects, including acute haemolytic reactions, infective contamination, and transfusion-related acute lung injury [6]. It is postulated that tranexamic acid (TXA) usage decreases blood loss in hip fracture patients and, thus, decreases the need for postoperative blood transfusion [7]. 

TXA is an anti-fibrinolytic agent, which is commonly used in obstetrics and polytrauma patients suffering from major haemorrhage [8]. It reversibly blocks lysine-binding sites on plasminogen molecules, thus reducing the conversion of plasminogen to plasmin and preventing clot dissolution.

There is evidence to support the use of TXA in haemorrhagic trauma patients [9]. There is emerging evidence to support the use of TXA in hip fracture surgery [10]. The current study aims to further the knowledge base around the use of TXA in hip fracture surgery patients. This study will focus on outcomes following the use of perioperative TXA in hip fracture patients, with the primary aim of assessing transfusion rates and thromboembolic events.

This article was previously presented as a meeting abstract at the Sylvester O'Halloran Conference, on March 4th, 2023. 

## Materials and methods

This was a single-centre, retrospective cohort study performed in a high-volume academic trauma unit managing 416 hip fractures per year. Patients undergoing surgery for a hip fracture between August 1, 2019, and August 1, 2020, were included.

Inclusion criteria were patients who received surgery after sustaining a hip fracture from August 2019 to August 2020. Exclusion criteria were patients who did not undergo surgery. Patients who received perioperative TXA were retrospectively identified as the treatment group. A control group consisted of hip fracture patients in the same period who did not receive TXA. There were 178 in the control group and 173 in the TXA group. On August 2, 2019, our institution underwent a policy change, whereby all hip fracture patients were given perioperative TXA unless a contraindication was evident, but ultimately, it was surgeon preference that determined who did and did not receive TXA.

Data sources

The local electronic registry in our institution was used to identify suitable participants. Patient data on preoperative and postoperative haemoglobin levels were collected from the electronic patient reporting system. Data regarding postoperative transfusion were retrieved from the National Transfusion Laboratory records. Data on patient medication before the hip fracture, with a particular focus on anti-coagulation medication, were collected by examining the anaesthetic notes as well as online patient records. Intraoperative blood loss was collected from intraoperative records when recorded. Data on symptomatic deep venous thrombosis (DVT) and pulmonary embolism (PE) were collected using the radiology system. Radiology reports examined included duplex Doppler studies for the presence or absence of DVTs, and computed tomography pulmonary angiography was used to determine the presence or absence of PEs. Venous thromboembolic (VTE) events were recorded as binary variables.

Variables

Patient records were reviewed for clinical and demographic variables of relevance. Patient age, sex, anti-coagulation medication prior to surgery, procedure type, and the American Society of Anaesthesiologists (ASA) Score were collected.

Data analysis

The primary outcomes of transfusion rate and VTE rate were compared between the TXA and control groups. Given the retrospective nature of this study, associations between variables were identified through the use of generalised linear models, which were able to produce risk ratios (RR) for each outcome variable of relevance. Confounder variables were identified and controlled for using multivariate regression analysis. Where appropriate, the relationship between two categorical variables was analysed using the chi-squared (χ²) test, provided there were more than five subjects in each group; otherwise, the Fisher’s exact test was used for these analyses. To determine the impact of categorical independent variables on interval dependent variables, the two-sample t-test with equal variances was used. A p-value of 0.05 was taken to be statistically significant, and the statistical software used was Stata/IC 13.1 for Mac (64-bit Intel; StataCorp LLC, College Station, TX, USA).

## Results

Over the study period, 416 hip fracture operations were performed, and 351 patients were eligible for inclusion in the study. Of these, 65 patients were excluded due to a lack of data reported on TXA administration.

Demographics

The mean age of the TXA group was 77.1 (SD ± 16.2), and the mean age of the control group was 79.2 (SD ± 16.1). The TXA group was 67% (n = 115) female, compared to 65.7% (n = 115) female in the non-TXA group (p = 0.795). A total of 173 (49.29%) patients were included in the TXA group (treatment group) and 178 (50.71%) in the control group. The SVUH hospital policy for TXA administration is 15 mg/kg prior to surgical induction. The most common dose of TXA administered was 1 g (80.9% of patients, n = 140). Other doses administered were 0.5 g (0.61%, n = 1), 0.75 g (6.75%, n = 11), 0.8 g (0.61%, n = 1), 0.9 g (3.07%, n = 5), 0.95 g (0.61%, n = 1), 1.25 g (0.61%, n = 1), and 1.5 g (1.84%, n = 3).

Of clinical relevance, 12.8% (n = 45) of the total population were taking regular antiplatelet medication, and 19.9% (n = 70) were taking anti-coagulation medication, with 15 patients with missing data not included (Tables 1-2).

**Table 1:** Patient demographics

Variable	Frequency
Sex, n (%)	
Male	119 (33.62)
Female	235 (66.38)
Age (years)	
Mean	78.2 (SD ± 16.7)
American Society of Anaesthesiologists (ASA) grade, n (%)	
1	14 (4.31)
2	138 (39.66)
3	178 (51.15)
4	17 (4.89)
Preop haemoglobin (g/dL)	
Mean	12 (SD ± 1.7)
Surgery category	
Blade plate	1
Cannulated screw	8
Dynamic hip screw	22
Hemiarthroplasty	159
Long intra-medullary nail	21
Short intra-medullary nail	110
Total hip arthroplasty	33

**Table 2:** Anticoagulation medication

Antiplatelet	Frequency	Percentage of cohort
Apixaban (2.5 mg BD)	10	2.82
Apixaban (5 mg BD)	6	1.69
Edoxaban (30 mg OD)	6	0.02
Edoxaban (60 mg OD)	2	0.56
Rivaroxaban (10 mg OD)	1	0.28
Rivaroxaban (15 mg OD)	6	1.69
Rivaroxaban (20 mg OD)	7	1.98
Warfarin (4 mg OD)	1	0.28
Warfarin (6 mg OD)	4	1.13
Warfarin (7 mg OD)	2	0.56
Total	45	12.71

Intraoperative transfusion

Sixteen patients (4.6%) required an intraoperative transfusion. Two variables were associated with intraoperative transfusion: ASA (11), analysed as a continuous variable (RR 2.97, 95% CI 1.38-6.35, p = 0.005), and preoperative haemoglobin levels (RR 0.91, 95% CI 0.88-0.94, p < 0.0001). After controlling for confounder variables in multivariate analysis, it was found that only preoperative haemoglobin was significantly associated with intraoperative transfusion rates (p < 0.0001).

Postoperative transfusion

**Figure 1:**
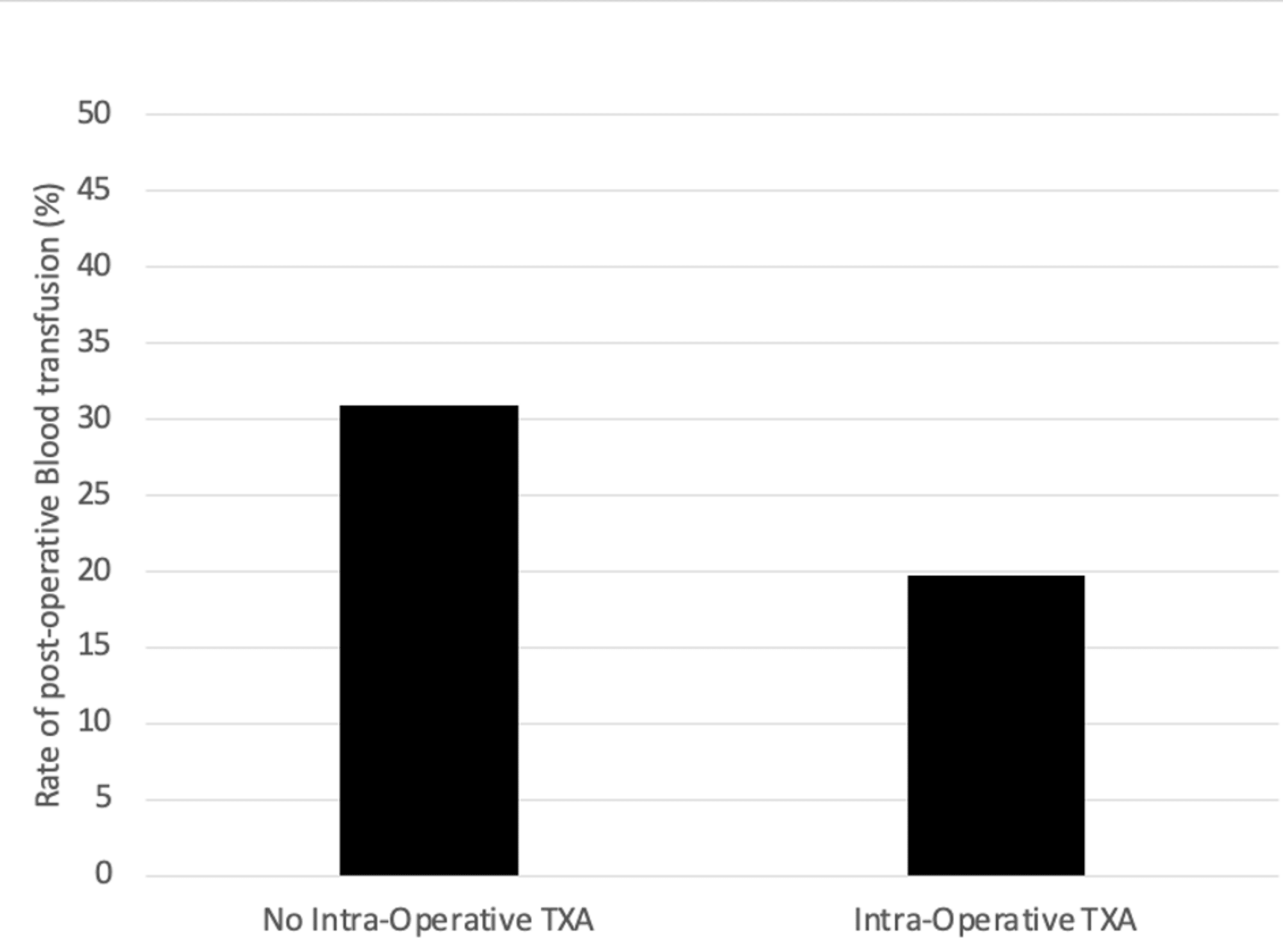
Rate of post-operative blood transfusion (TXA vs no TXA) A total of 89 patients required blood transfusion: 55 (62%) had not received TXA, and 34 (38%) did receive TXA (p = 0.022). TXA, tranexamic acid

During the study period, 89 patients (25.1%) required postoperative transfusion. Of these 89 patients, 55 (62%) had not received TXA, and 34 (38%) did receive TXA (Figure 1). On univariate analysis, there were four variables associated with postoperative transfusion: age (RR 1.03, 95% CI 1.01-1.04, p < 0.0001), ASA (RR 1.75, 95% CI 1.35-2.27, p < 0.001), TXA (RR 0.64, 95% CI 0.45-0.94, p = 0.022), and preoperative haemoglobin level (RR 0.93, 95% CI 0.90-0.94, p < 0.0001). After controlling for confounder variables in multivariate analysis, it was found that both preoperative haemoglobin (p < 0.0001) and administration of TXA (p = 0.047) were significantly associated with a reduced need for postoperative transfusions. The use of regular anticoagulant (p = 0.12) and antiplatelet (p = 0.52) medication was not associated with higher postoperative transfusion rates.

Postoperative haemoglobin

Day 1 haemoglobin levels in the TXA group were significantly higher compared to the no-TXA group (107 g/L, s = 19.9, 95% CI 103.1-109.8 vs 101 g/L, s = 19.2, 95% CI 97.9-104.1, p = 0.0183). There were two variables associated with a reduced postoperative transfusion after univariate analysis: the administration of TXA (p = 0.018) and preoperative haemoglobin (p < 0.0001). On multivariate analysis, it was found that both preoperative low haemoglobin (p < 0.0001) and lack of TXA administration (p = 0.047) were associated with increased rates of postoperative transfusions.

Venous thromboembolism (VTE)

With regard to postoperative VTE, two patients sustained a DVT, and two sustained a PE. All DVT and PE cases were in the TXA group (DVT, p = 0.242; PE, p = 0.242), indicating no statistical significance. There was also no significant association between the use of anticoagulant or antiplatelet agents and the rates of VTE (antiplatelet and DVT, p = 0.36; antiplatelet and PE, p = 1.0; anticoagulation and DVT, p = 1.0; anticoagulation and PE, p = 1.0).

## Discussion

It has been demonstrated that increased blood loss and subsequent transfusions may have a significant impact on hip fracture patients, particularly those who are elderly with multiple co-morbidities [11]. We demonstrate the importance of TXA administration, resulting in an associated reduction in postoperative transfusion rates and improved Day 1 haemoglobin levels, as shown by multivariate analysis. This reduction in transfusion frequency is consistent with recent studies in the area [7,10]. Although our study demonstrated that preoperative haemoglobin levels are also an important predictive factor, this is a non-modifiable factor, so it is helpful to identify that the only modifiable factor of significance is the administration of TXA.

In addition, this study illustrated that the use of TXA is not associated with an increase in the risk of thromboembolic events postoperatively. This is in contrast to a recent study by Viberg et al., which illustrated that the use of TXA in a hip fracture patient population decreases the rate of VTE postoperatively [10]. This study encompassed a total of 3,097 patients, and results demonstrated a reduction in the risk of thromboembolic events after 30 days, with a RR of 0.63 [10]. Also of note, as a result of similar findings in the CRASH-2 Trial, there is a hypothesis that TXA might have an anti-inflammatory effect, inhibiting the inflammatory effects of plasminogen and plasmin [9,12,13]. Despite this documentation, our study did not demonstrate an association with a decrease in the rates of postoperative VTE. Overall, the outcome of a neutral effect on VTE events supports our hypothesis and aligns with the current literature regarding the safety profile of TXA use in hip fractures [10].

Hip fracture surgeries are associated with risks of bleeding, as well as VTE events. Concurrently, anticoagulants are effective prophylaxis for VTE events, but they simultaneously increase the risk of bleeding. A double-blinded, randomised, and placebo-controlled trial provided evidence that, despite regular anticoagulation use, the use of TXA is still beneficial in reducing postoperative transfusion rates [14]. Our study also illustrated that, despite regular anticoagulation, TXA is associated with reduced postoperative transfusion rates.

Limitations

This current study has a number of limitations. It was a retrospective study and thus prone to bias. The sample size is relatively small, so we may be underpowered to detect subtle outcomes associated with TXA on a larger scale. The patient cohort examined in this study was not randomised; a higher-powered, randomised controlled trial would be beneficial in further assessing the efficacy of TXA use in this patient population. At the time of this study, there was no standardised protocol to determine the timing and dose of TXA for patients; this lack of standardisation may affect the interpretability of some findings. This study did not monitor for asymptomatic PE or DVT, so these events could have been undetected or treated elsewhere outside the institution’s hospital.

## Conclusions

Administering intraoperative TXA is associated with reduced postoperative transfusion rates and improved postoperative haemoglobin levels in hip fracture patients, while not increasing VTE events. Further research in this field should focus on determining an ideal dose, mode of delivery, and timing of TXA administration to optimise its efficacy.
